# Identification of a putative new therapeutical target for Hodgkin's lymphoma

**DOI:** 10.1186/2194-7791-2-S1-A24

**Published:** 2015-07-01

**Authors:** Stefanie Kewitz, Ines Volkmer, Martin S Staege

**Affiliations:** Department of Pediatrics, Martin Luther University Halle-Wittenberg, Ernst-Grube-Str. 40, 06097 Halle, Germany

## Aims

Hodgkin's lymphoma (HL) is a malignant disease of the lymphatic system. In children, the 5 year event-free survival rate is approximately 90%. However, the currently used therapy is associated with severe late side effects. Reduction of treatment intensity might reduce the risk for late toxicities but might also increase the risk for relapses. Therefore, it is important to identify new targets for improvement of the current therapy and development of new treatment strategies.

## Methods

We established cDNA libraries from chemoresistant HL cell lines and developed a functional screening approach for the identification of genes which are potentially involved in the resistance against cytostatic drugs. For this end, we transfected the libraries into cells, treated them with cisplatin, re-isolated and sequenced the vectors from surviving cells.

## Results

One of the isolated vectors contained the cDNA of a protein kinase form chromosome 6. Expression analysis showed that this kinase is only expressed in HL cell lines and other cancer cell lines (*e.g*. from Ewing sarcoma, neuroblastoma and leukemia) but not in normal blood cells or in normal tissues, except testis. Knock-down experiments and the incubation with an inhibitor of the kinase confirmed that this new factor modulates the chemosensitivity of HL cells. We used DNA-Microarray analyses and identified genes which were regulated by the kinase. Furthermore we isolated new interaction partners of the kinase and analyzed whether the kinase can be used as new target for immunotherapy.

## Conclusion

Our data indicate that the identified kinase is associated with the resistance of the cells against cytostatic drugs and might be an interesting target for the development of new treatment strategies for patients with Hodgkin's lymphoma.

